# Fabrication and Characterization of Semi-Resorbable Bioactive Membrane Derived from Silk Fiber Sheet for Guided Bone Regeneration

**DOI:** 10.3390/biomimetics10110790

**Published:** 2025-11-20

**Authors:** Kanokporn Santavalimp, Jirut Meesane, Juthakarn Thonglam, Kawintip Prasongyuenyong, Prisana Pripatnanont

**Affiliations:** 1 Cranio-Maxillofacial Hard Tissue Engineering Center, Oral and Maxillofacial Surgery Section, Faculty of Dentistry, Prince of Songkla University, Hat Yai 90110, Songkhla, Thailand; kanokporn.san@psu.ac.th (K.S.); kawintip.p@psu.ac.th (K.P.); 2 Institute of Biomedical Engineering, Department of Biomedical Science and Biomedical Engineering, Faculty of Medicine, Prince of Songkla University, Hat Yai 90110, Songkhla, Thailand

**Keywords:** barrier membrane, biphasic calcium phosphate, guided bone regeneration, polyvinyl alcohol, silk fiber sheet, silk fibroin

## Abstract

The barrier membrane is a key component in guided bone regeneration (GBR); however, there is no current commercially available membrane universally suitable for all clinical situations. The semi-resorbable bioactive barrier membrane derived from a silk fiber sheet (SF), polyvinyl alcohol (PVA), and biphasic calcium phosphate (BCP) was fabricated to provide improved physical, mechanical, and bioactive properties. There were four experimental groups: PVA/SF, 1BCP/PVA/SF, 3BCP/PVA/SF, and 5BCP/PVA/SF. All fabricated membranes appeared white in color with a smooth texture; however, SEM images revealed a rougher top surface compared to the bottom surface. FTIR and DSC validated the presence of the SF and PVA with or without BCP. All membranes displayed high hydrophilicity, except the PVA/SF group, which remained hydrophobic on the bottom surface. The water uptake of all groups reached the plateau phase within 10 min. The degradation rate fell within the range of 5–20% over a three-month period. Both fibroblastic and osteoblastic cells attached and survived on the BCP-incorporated membranes, comparable to those observed in the commercially available ossifying collagen membrane. Among the fabricated membranes, the 3BCP/PVA/SF formulation demonstrated the most favorable physical, mechanical, and biological properties for GBR applications.

## 1. Introduction

Guided bone regeneration (GBR) utilizes a membrane with a barrier function to prevent fast-growing epithelial and connective tissues in the grafted site and to help bone-forming cells to regenerate [1]. The commercially available barrier membranes are categorized into non-resorbable and resorbable types. Non-resorbable membranes, mainly expanded polytetrafluoroethylene (e-PTFE), demonstrate biocompatibility and a space maintenance ability, making them suitable for large or non-contained bony defects. However, because these membranes do not naturally degrade in the human body, a second procedure is required for their removal. Resorbable membranes eliminate the need for a membrane removal procedure but may lack long-term barrier functions [2]. To enhance the functional performance of the membranes, natural elements or synthetic bioactive materials can be incorporated along with biological factors [2,3].

Silk fibroin (SF) is a fibrous protein extracted from natural fiber with a semi-crystalline structure that has been widely used in various biomedical applications. It can be manufactured into a GBR membrane through various fabrication techniques [4], such as solvent casting [5], lyophilization, densification, ethanol treatment [6], and electrospinning [7].

A semi-resorbable barrier membrane was fabricated from an SF solution mixed with glycerol as a plasticizer, incorporated with fish collagen. The membrane exhibited a thin, film-like structure with surface micro-roughness and an absence of porosity on both sides. However, it demonstrated good biocompatibility in vitro and in vivo. In comparison to commercially available resorbable collagen membranes, it provided a more prolonged barrier effect and remained structurally stable without collapsing and promoted more bone formation [8,9]. Although the SF solution-based membranes demonstrated good outcomes, the use of the SF solution combined with glycerol still limited the mechanical strength.

Silkworms produce silk by secreting a protein called fibroin from their salivary glands, and contact with air hardens the protein into a continuous filament. When the silkworms are restricted to spin on a flat surface, they produce a silk fiber sheet: a paper-like cocoon with enhanced mechanical properties and high tensile strength [10]. This material shows promising properties as an alternative base for semi-resorbable GBR membranes. However, the major limitation of the silk fiber sheet is its hydrophobic nature, which negatively impacts the cellular activities of tissue regeneration. To address this, additional synthetic or natural materials can be incorporated, including polyvinyl alcohol (PVA). PVA is a biodegradable synthetic polymer with low toxicity and is generally used as a polymeric binder for composite biomaterials. The addition of PVA to a silk fibroin membrane improves its physical properties by increasing hydrophilicity and potentially enhances the cellular biocompatibility for further biomedical applications [11,12,13].

Incorporating synthetic bioactive ceramics is a potential modification strategy to provide mechanical support and supply a sustained calcium source. Biphasic calcium phosphate (BCP), composed of hydroxyapatite (HA) and β-TCP, is one of the most commonly used synthetic bioceramics in bone regeneration due to its excellent bioactivity and high mechanical strength [14], making it a promising candidate for modifying the barrier membrane.

This study aims to fabricate and characterize a novel conceptual “semi-resorbable bioactive barrier membrane” derived from a silk fiber sheet combined with PVA and BCP. The BCP component is designed to promote bone formation, while the overall material is expected to be semi-resorbable and provide sufficient space maintenance for bone regeneration. After the barrier function ceases due to the degradation of the PVA polymeric binder, the BCP bioactive component may further support new bone formation and integrate with surrounding tissue without the need for a second removal surgery.

## 2. Materials and Methods

This study comprised 2 parts: Part I—fabrication of the semi-resorbable bioactive barrier membranes derived from silk fiber sheet—and Part II—characterization of the fabricated membranes in respect to physicochemical, mechanical, and biological properties and comparison with the commercial ossifying collagen membrane (Ossix^®^ Plus, Datum Dental Ltd., Yavne, Israel). This membrane is a porcine type I collagen-based sugar-crosslinked membrane that can ossify and provide a long-term barrier effect for 4–6 months.

## 3. Part I: The Fabrication of the Semi-Resorbable Bioactive Barrier Membranes

### 3.1. Preparation of Silk Fiber Sheet

Silk fiber sheets derived from *Bombyx mori* silkworms were prepared by the Office of Sericulture Research and Development, the Queen Sirikit Department of Sericulture, Thailand. The sheets were cut into 3 × 3 cm^2^ squares and mounted on plastic frames. The degumming process was performed in 0.02 M Na_2_Co_3_ (Sigma-Aldrich, St. Louis, MO, USA) at 80 °C for 15 min. The sheets were then rinsed with distilled water and stored at −80 °C for 1 h and then freeze-dried for 12 h to remove excess moisture.

### 3.2. Preparation of BCP-PVA Suspension

PVA (Sigma-Aldrich, MO, USA) was dissolved in deionized water (3% *w*/*v*) at 90 °C; then BCP (HA:β-TCP = 30:70) (the National Metal and Materials Technology Center (MTEC), Pathum Thani, Thailand) was added to the PVA solution at varying concentrations (0, 1%, 3%, and 5% *w*/*v*) and ultrasonically stirred for 1 h for uniform dispersion and then left at room temperature to defoam.

### 3.3. The Preparation of the Semi-Resorbable Bioactive Barrier Membranes

**Figure 1:**
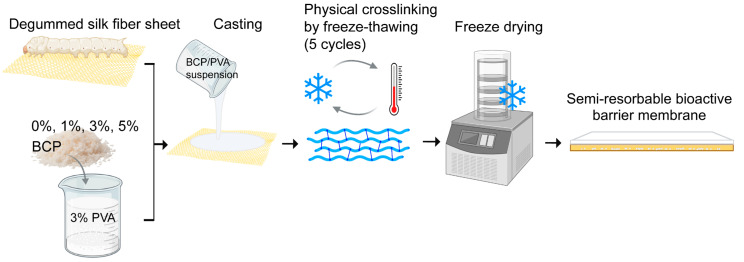
The process diagram of the membrane construction for a semi-resorbable bioactive barrier membrane derived from a silk fiber sheet for guided bone regeneration.

To fabricate the semi-resorbable bioactive barrier membranes, BCP-PVA suspension (0, 1%, 3%, and 5% *w*/*v*) was cast onto a degummed silk fiber sheet in a plastic template; then it underwent freeze–thawing for five cycles by freezing at −80 °C for 1 h and thawing at room temperature for 4 h per cycle. Thereafter, each sample was stored overnight at −80 °C and freeze-dried for 24 h to remove excess water (Figure 1).

## 4. Part II: The Characterization of the Semi-Resorbable Bioactive Barrier Membranes

The fabricated membranes were classified into four experimental groups according to the concentration of BCP in the PVA suspension. The commercial ossifying collagen membrane was used as the control group. Abbreviations and percentages of BCP of each group are presented in Table 1.

### 4.1. The Physicochemical Characterization of the Semi-Resorbable Bioactive Barrier Membranes

#### 4.1.1. Gross Observation

The membranes were grossly inspected for their physical appearance in terms of color, flexibility, and texture. The average membrane thickness was measured using digital vernier calipers (Mitutoyo, Kawasaki, Japan) and reported as mean ± SD (*n* = 5).

#### 4.1.2. Scanning Electron Microscope (SEM)

The surface and cross-section structure of the membranes were inspected using SEM (Quanta400, FEI, Brno, South Moravian Region, Czech Republic). The pore size and surface roughness were inspected from SEM images by ImageJ software Version 1.54 (Java program, San Francisco, CA, USA) and described as means and SD.

#### 4.1.3. Fourier Transform Infrared Spectroscopy (FTIR)

FTIR (Bruker EQUINOX 55, Germany) was used to detect the chemical composition of the fabricated membrane. The wave numbers ranged from 400 to 4000 cm^−1^ using the KBr disk technique with Attenuated Total Reflectance mode. The Opus 3.0 data collection software program (Bruker, Ettlingen, Germany) was used for spectral data analysis.

#### 4.1.4. Differential Scanning Calorimetry (DSC)

The DSC instrument (DSC7, Perkin Elmer, Waltham, MA, USA) was used to investigate the thermal decomposition of the membranes. The temperature ranged from 20 °C to 350 °C, at 5 °C/min under a flow of nitrogen.

#### 4.1.5. Water Contact Angle

A static water contact angle was used to determine the wettability of the membranes. After each membrane was fixed to a metal plate, 2 μL of deionized water was dropped from 1 mmonto its surface, under ambient humidity. Five points in different locations were used to measure the angle formed by the liquid drop on the surfaces using a contact angle meter (KYOWA, Tokyo, Japan).

#### 4.1.6. Swelling Degree (%)

The fabricated membranes were trimmed to a size of 10 mm × 10 mm (*n* = 5) and then immersed in a phosphate-buffered saline solution (PBS) with a pH of 7 at 37 °C. The weights of each membrane were recorded before and after soaking in the PBS for 2, 5, 10, 15, and 30 min, then 1, 2, 4, 6, 12, and 24 h. The swelling degree at the predetermined time was calculated using the following formula:

Swelling degree (%) = [(Vt − Vo)/Vo] × 100

where

Vo = initial weight of dry membrane;
Vt = final weight of the swollen membrane at the predetermined time.

#### 4.1.7. Degradation Rate (%)

The membranes were trimmed to a size of 10 mm × 10 mm (*n* = 5) and then soaked in PBS solution containing lysozyme (2 μg/mL) at pH 7.4 for 1, 2, 4, 6, 8, and 12 weeks at 37 °C. Thereafter, the residual membrane was frozen at −80 °C for 24 h and freeze-dried to remove the excess solution. The degradation rate at the predetermined time was calculated using the following formula:

Degradation rate (%) = [(Wt − Wo)/Wo] × 100

where

Wo = initial weight of dry membrane;
Wt = final weight of the remaining membrane at the predetermined time.

#### 4.1.8. The Calcium Release Profile of the Membranes

The membranes were trimmed to sizes of 10 mm × 10 mm (*n* = 5) and then immersed in 100 μm of ultra-pure distilled water at 37 °C for 1 h and then for 21 days. At each time point, one aliquot was removed for calcium content analysis by Calcium Colorimetric Assay kit (MAK022-1KT Calcium Colorimetric Assay, Sigma-Aldrich, MO, USA). The absorbance was quantified at 570 nm, and the data were converted into concentration by means of a standard curve.

### 4.2. The Mechanical Characterization of the Semi-Resorbable Bioactive Barrier Membranes

The mechanical characteristics of the fabricated membranes were evaluated using a universal testing machine (LRX-Plus, AMETEK Lolyd Instrument Ltd., Hampshire, UK). Five membranes of each experimental group were trimmed into rectangular shapes with a size of 10 mm × 30 mm and then secured with acrylic jigs at both ends. Tensile force was applied at a speed of 10 mm/min continuously to obtain a maximum load–maximum elongation graph. Data from the graph was used to calculate the tensile strength, percentage of elongation, and Young’s modulus of the membranes.

### 4.3. The Biological Characterization of the Semi-Resorbable Bioactive Barrier Membranes

#### 4.3.1. Cell Culture

The fibroblast L929 cell lines (ATCC, Manassas, VA, USA) were cultured in Dulbecco’s Modified Eagle Medium (DMEM) containing 10% fetal bovine serum (FBS), 1% penicillin/streptomycin, and 0.1% amphotericin B (GibcoTM, Invitrogen, Thermo Fisher Scientific, Waltham, MA, USA). The pre-osteoblast MC3T3-E1 cell lines (ATCC, Manassas, VA, USA) were cultured in the alpha-minimum essential medium (α-MEM) containing 10% FBS, 1% penicillin/streptomycin, and 0.1% amphotericin B. The cells were cultured at 37 °C in a humidified atmosphere containing 5% CO_2_. The medium was changed every 3 days until confluence and then subcultured for the experiments.

#### 4.3.2. Cell Attachment and Morphology

The membranes were trimmed to a size of 10 × 10 mm^2^ and placed into a 24-well plate filled with the culture medium. The L929 cells were seeded on the top surface, while the MC3T3-E1 cells were seeded on the bottom surface of the membrane at a density of 2 × 10^4^ cells/cm^2^, separately, and cultured for 6 and 24 h. Thereafter, each membrane was removed, washed with PBS, and fixed in 10% formaldehyde (Sigma Aldrich, St. Louis, MO, USA) for 30 min, then freeze-dried. The cell-cultured membranes were secured on metal studs and coated with gold for cell attachment and morphology investigation via SEM (Quanta400, FEI, Brno, South Moravian Region, Czech Republic).

### 4.4. Statistical Analysis

The data were analyzed using statistical analysis software (GraphPad Prism, Version 10.4.1, USA). The Shapiro–Wilk normality test was applied. The normally distributed data employed a One-Way Analysis of Variance (ANOVA) followed by Tukey’s HSD test to detect the differences among the groups. Repeated measures of ANOVA and paired *T*-tests were applied to determine the differences at each time point. The statistical significance was set at a *p*-value of <0.05. The Kruskal–Wallis test was used for non-normal distribution data. Descriptive analysis was used for physicochemical and biological characterization.

## 5. Results and Discussion

The semi-resorbable bioactive barrier membranes derived from silk fiber sheets combined with PVA and incorporated with BCP were successfully fabricated via casting and freeze-drying methods, which is simple and suitable for the manufacture of the biomaterials in large volumes [15]. The BCP-incorporated membranes demonstrated a calcium-ion-releasing property, which can be precipitated in grafted sites and induce bone formation [16,17,18].

### 5.1. The Physicochemical Characterization of the Semi-Resorbable Bioactive Barrier Membranes

#### 5.1.1. Gross Observation

**Figure 2:**
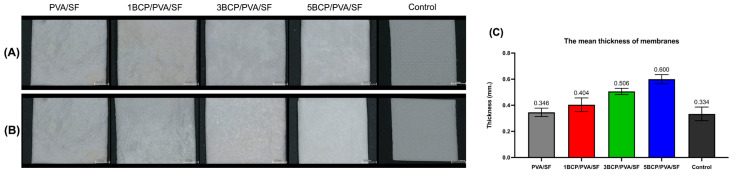
Gross specimens of the membranes under the stereomicroscope: (**A**) the top surface of the membranes, (**B**) the bottom surface of the membranes (Scale bar = 2 mm), and (**C**) the mean thickness of the membranes.

The fabricated membranes were white and flexible. The surfaces were smooth under direct observation, but the top surface had a rougher texture than the bottom surface under a stereomicroscope (Figure 2A). The mean thickness of the membranes ranged from 0.3 to 0.6 mm.

The thickness of the membranes may influence their handling characteristics and suitability for application during surgical procedures. The mean thickness (Figure 2B) of the fabricated membranes was slightly thicker than the control group (a sugar crosslink resorbable collagen membrane) and also thicker than the d-PTFE membrane (0.13 to 0.25 mm) [19]. However, the thick barrier membrane may prolong its barrier function and enhance the bone regeneration capability. Bubalo M et al. demonstrated that the placement of a thick human resorbable demineralized collagenous membrane reduces soft tissue ingrowth and promotes superior bone healing in extraction sockets in dogs [20].

#### 5.1.2. Scanning Electron Microscope (SEM)

**Figure 3:**
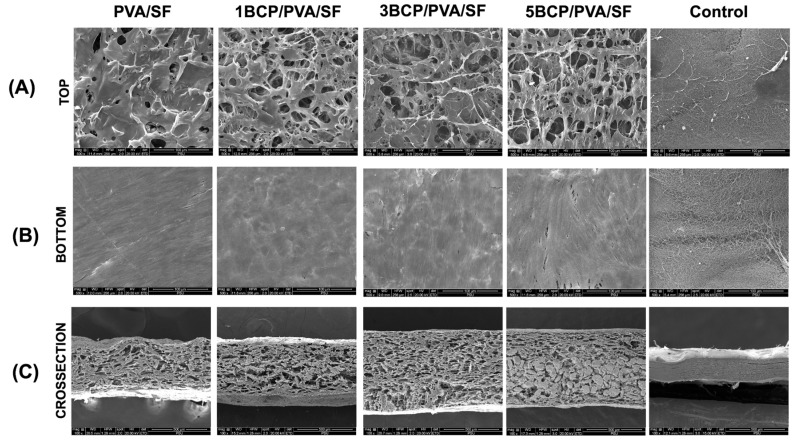
SEM images of the fabricated membranes: (**A**) the top surface images at a magnification of ×500, (**B**) the bottom surface images at a magnification of ×500, and (**C**) the cross-sectional images at a magnification of ×100.

From the SEM images, the membranes showed different patterns on each side, as shown in Figure 3. The fabricated membrane demonstrated a smooth surface without porosity on the bottom surface, while the top surface demonstrated the diffusion of pore structures. The cross-sectional images demonstrated a uniformly porous structure throughout the membranes.

**Figure 4:**
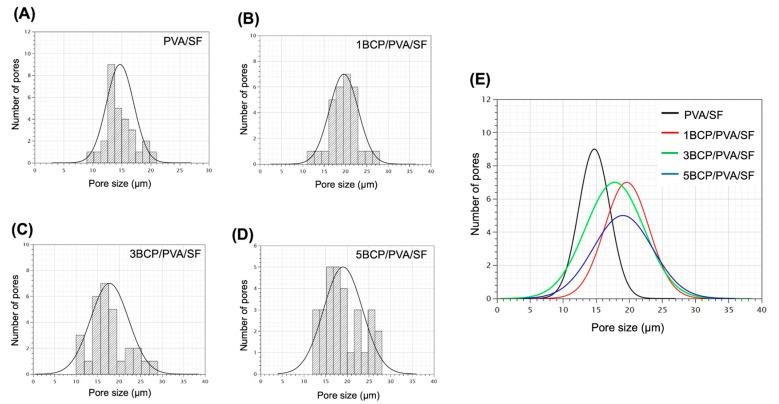
Pore size distribution histograms of (**A**) PVA/SF, (**B**) 1BCP/PVA/SF, (**C**) 3BCP/PVA/SF, and (**D**) 5BCP/PVA/SF. (**E**) Mean pore size of all samples.

The mean pore size of the top surface of each group is presented in Figure 4. The average pore size of all groups is in the range of 10–30 μm, with no statistically significant differences between groups.

**Figure 5:**
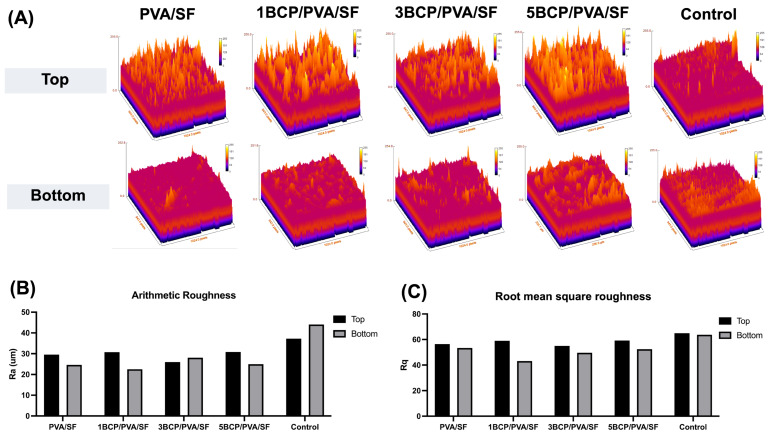
(**A**) The 3D surface plot of SEM images of the top surface (upper row) and the bottom surface (lower row) using the ImageJ software, (**B**) the arithmetic mean roughness (Ra) of the fabricated membranes, and (**C**) the root mean square roughness (Rq) of the fabricated membranes.

The surface roughness was determined using a 3D surface plot of SEM images by ImageJ software (Figure 5). The top surface of the fabricated membrane was slightly rougher than the bottom surface. The arithmetic mean roughness (Ra) of the fabricated membrane was around 20–30 μm on both sides, which is less than the control (40–45 μm). For the root mean square roughness (Rq), the bottom surface of the fabricated membrane had a slightly smaller Rq than the top surface; meanwhile, there was no significant difference between the two sides of the control membrane. Furthermore, the BCP-incorporated membranes showed a more irregular surface and increased roughness with the increased ratio of BCP.

The different surface architecture of the fabricated membrane, as observed under SEM indicated the side specificity of the membrane. The bottom surface had a smooth architecture with diffuse BCP particles, caused by the casting of the PVA solution or the BCP/PVA suspension onto a silk fiber sheet in the template, and then the BCP particles likely settled toward the bottom surface during the freeze–thaw process. The bottom surface was compatible with the hard-tissue-facing side, enhancing bone formation and acting as a bone scaffold. The top side was intended to face the soft tissue due to its rough and porous structure, which promotes cell attachment and facilitates wound healing. The porous structure facilitates the migration and proliferation of the cells by establishing appropriate conditions for nutrient exchange throughout the membranes [21].

The barrier membrane with the proper porous structure enables the cell occlusive properties to work while accelerating vascularization and allowing nutrients to access the grafted site [22]. For the non-resorbable membrane, e-PTFE (Gore-Tex^®^) is composed of a large pore (100–300 μm porosity), in order to promote an ingrowth of collagen to enhance membrane stability, and an occlusive pore (<8 μm porosity), for blocking soft tissue ingrowth [23,24]. For the d-PTFE membrane, a non-expandable, high-density, and low porosity (0.2 to 0.3 μm) material resists bacterial contamination even when exposed to the oral cavity [19,25,26]. Nevertheless, the microporous range of 0.2–0.3 μm jeopardizes the blood supply to the underlying area.

A natural resorbable collagen membrane demonstrates a wide range of porosities, from microporosity to microporosity. The pores of 25–50 μm are suitable for angiogenesis, while the microporous (50–100 and 100–150 μm) areas are suitable for bone and soft tissue ingrowth [27]. The barrier membrane should contain pore sizes that do not exceed 100 μm to block the invasion of soft tissue into the grafted area [28]. In this study, the pore size of the top surface of the fabricated membrane was in the range of 10–30 μm, which provided physical barrier functions to the fibroblast cells. Then, during the degradation process, the pore size may open and allow the passage of cells and vascularization, which occurs at ≥30–40 μm.

#### 5.1.3. Fourier Transform Infrared Spectroscopy (FTIR)

**Figure 6:**
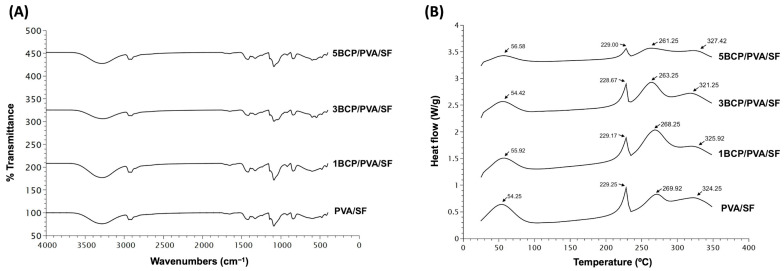
(**A**) Fourier transform infrared spectroscopy (FTIR) spectra of the fabricated membrane in the wavelength range of 400–4000 cm^−1^ and (**B**) Differential Scanning Calorimetry (DSC) thermogram of the fabricated membrane from 20 °C to 350 °C.

The chemical composition of the fabricated materials is shown in Figure 6A. All fabricated membranes showed the FTIR spectrum of the base materials of silk fibroin, with OH stretching, and the amide III of the β-sheet structure. The intensity peak of 3200–3500 cm^−1^ indicated −OH stretching. Amide I (C=O stretching) was seen at 1620–1635 cm^−1^, amide II (N-H bending) was shown at 1515–1530 cm^−1^, and amide III (C-N stretching or N-H bending) was found at 1219–1313 cm^−1^, which indicated the β-sheet structure. Random coils were found at 1640–1648, 1535–1545, and 1235 cm^−1^, respectively [29].

The FTIR spectra of the PVA related to the hydroxyl and acetate groups. Large bands were observed between 3550 and 3200 cm^−1^ and linked to the O–H stretching from the intermolecular and intramolecular hydrogen bonds. The vibrational band was between 2840 and 3000 cm^−1^ and referred to the C–H stretching from alkyl groups, and the peaks between 1750 and 1735 cm^−1^ were due to the stretching of C=O and C–O from the acetate group remaining from the PVA [30].

The fabricated membrane presented the bands related to PO_4_^3−^ (560 and 600 cm^−1^), CO_3_^2−^ (1000–1100 cm^−1^), HPO_4_^2−^ (570 cm^−1^, 602 cm^−1^, and 963 cm^−1^), and the OH group. The weak peaks were found between 870 and 880 cm^−1^, and the intense peaks were between 1460 and 1530 cm^−1^. The BCP consists of β-TCP and HA, and the bands that were presented at 3571 cm^−1^ and 631 cm^−1^ represented a peak of the OH group in the HA phase. The three groups with incorporated BCP showed that the bands between 569 and 1040 cm^−1^ were specific bands for β-TCP. The peak intensity varied with the amount of remaining BCP in the membrane [31,32].

The FTIR confirmed the base of the mixture compound substrates of the PVA and SF with −OH stretching and amide III of the β-sheet structure, which contributed to the structural strengthening [33] of the silk fiber sheet in all groups. Apart from the spectrums of HAp and β-TCP, the peak shifts in the O–H stretching confirmed the physical bonding between BCP and PVA [31,34].

#### 5.1.4. Differential Scanning Calorimetry (DSC)

The thermal behavior of the membranes was observed by Differential Scanning Calorimetry (DSC) from 20 °C to 350 °C. Figure 6B shows four endothermic peaks in all groups of membranes. The first peak of PVA/SF (54.25 °C), 1BCP/PVA/SF (55.92 °C), 3BCP/PVA/SF (54.42 °C), and 5BCP/PVA/SF (56.58 °C) represented residual water in the materials [35].

The second, third, and fourth peaks were the melting temperatures of each base material: PVA/SF (229.25 °C, 269.92 °C, 324.25 °C), 1BCP/PVA/SF (229.17 °C, 268.25 °C, 325.92 °C), 3BCP/PVA/SF (228.67 °C, 263.25 °C, 321.25 °C), and 5BCP/PVA/SF (229.00 °C, 261.25 °C, 327.42 °C).

The melting peak temperatures in DSC were consistent with the pure PVA material (at 200–240 °C) [36] and pure SF. The decomposition temperature of SF begins around 260 °C to 290 °C, and the main degradation occurs at higher temperatures, typically peaking between 313 °C and 325 °C, varies depending on the silk’s structure, and is usually not more than 350 °C [37,38]. Since the membranes were fabricated based on the degummed silk fiber sheet, the thermal behavior of the membranes was observed by DSC from 20 °C to 350 °C to prove that the degummed process to remove sericin during the membrane fabrication still maintained the property of the silk fibroin protein. The thermal behaviors of these membranes were related to the FTIR results. The melting temperature tended to be lower when the percentage of BCP increased, which led to the irregular structural arrangement of the materials and the structural strength.

#### 5.1.5. Water Contact Angle

The water contact angle was used to determine the surface energy and surface wettability of materials. The material’s wettability is important for biomedical materials to be biocompatible and friendly to cell adherence on its surface [39].

On the top surface of the membranes, the water contact angles of the materials PVA/SF (65.79° ± 0.52), 1BCP/PVA/SF (80.01° ± 12.11), 3BCP/PVA/SF (88.25° ± 3.71), and 5BCP/PVA/SF (69.07° ± 3.92) were less than 90°, indicating the hydrophilic property of the membranes.

**Figure 7:**
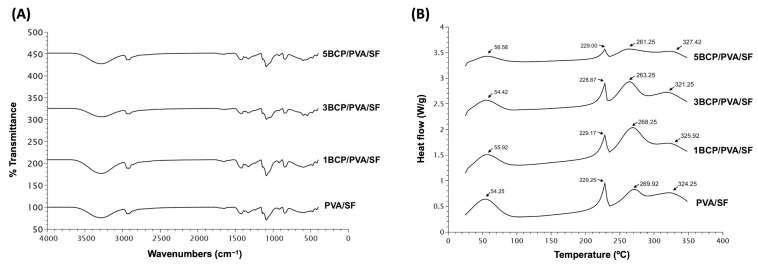
(**A**) The water droplet configuration on the top surface (upper row) and the bottom surface (lower row) of the membranes, and (**B**) the degree of the water contact angle on each side of the membranes.

On the bottom surface, only PVA/SF (99.16° ± 3.96) exhibited the hydrophobic property, while other membranes, including 1BCP/PVA/SF (66.89° ± 3.38), 3BCP/PVA/SF (62.49° ± 8.07), and 5BCP/PVA/SF (79.90° ± 4.9), were hydrophilic, as shown in Figure 7.

Silk fibroin comprises a hydrophilic light chain (≈25 kDa) called silk-I, which imbues elasticity to silk, and a hydrophobic heavy chain (≈350 kDa) or β-sheets, called silk-II, which provide the strength of the fiber. The ratio of silk-I and silk-II, and the preparation methods affect the final properties of the silk [40]. Regarding the FTIR analysis, the regenerated silk fibroin from the silk fiber sheet in this study represents the β-sheet structure, which possesses a hydrophobic property. This effect influenced the bottom surface of the PVA/SF membrane to be hydrophobic, even though PVA is a hydrophilic polymer [41]. However, the incorporation of BCP had a positive effect on the membrane and enhanced the hydrophilicity of the membrane to favor protein and cell attachment.

#### 5.1.6. Swelling Degree (%)

**Figure 8:**
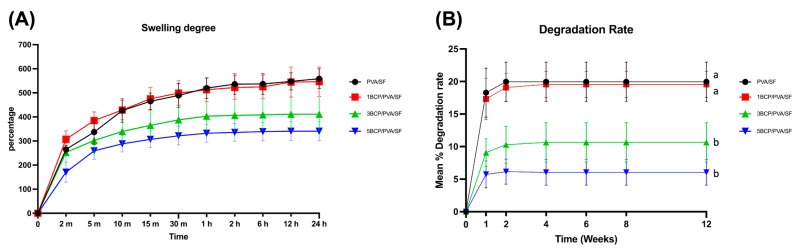
(**A**) The swelling degree of the fabricated membranes in PBS at 37 °C. (**B**) The degradation rate of fabricated membranes in the PBS solution containing lysozyme (2 μg/mL) at 37 °C (a = significant difference between 3BCP/PVA/SF and 5BCP/PVA/SF, and b = significant difference between PVA/SF and 1BCP/PVA/SF (*p* < 0.05)).

The swelling degree of each material in the PBS at 37 °C is shown in Figure 8A. The swelling rate rose rapidly in the first 2 min, then gradually increased up to 10 min in all groups. After the membrane saturation for 24 h, the swelling degrees of PVA/SF (558.44 ± 41.23%) and 1BCP/PVA/SF (566.35 ± 61.49%) were higher than 3BCP/PVA/SF (411.28 ± 68.75%) and 5BCP/PVA/SF (340.75 ± 39.37%), but statistically significant differences among groups were not detected.

The swelling degree represents the fluid uptake property of the material, which depends on the thickness, pore geometry, and porosity structure within the material. This property affects the materials’ mechanical properties and protein absorption, which leads to cell adhesion and proliferation on the membrane structure [42]. The fabricated membrane possessed the ability to absorb water, while still favoring the membrane handling during the surgery. Groups 3BCP/PVA/SF and 5BCP/PVA/SF showed inverse swelling pattern to the ratio of BCP, providing better structural stability.

#### 5.1.7. Degradation Rate (%)

The degradation rate of the tested samples in lysozyme is shown in Figure 8B. All samples in the control group were completely degraded in lysozyme within the first week, while all samples of the experimental groups remained yet rapidly degraded in the first week but then reached a stable plateau until the fourth week. After 12 weeks, the mean degradation rates of PVA/SF (19.97 ± 3.02%) were comparable to 1BCP/PVA/SF (19.56 ± 2%) and statistically significantly higher (*p* < 0.05) than 3BCP/PVA/SF (10.65 ± 3.01%) and 5BCP/PVA/SF (6.05 ± 1.98%).

The degradation rate affected the fabricated membranes as a “semi-resorbable” property according to the composition of the substrate materials: a water-soluble PVA [43] and proteolytic enzymatic soluble silk fibroin that degrades from days to years in vivo [44]. However, the fabricated materials in this study, derived from a silk fiber sheet with a natural electrospun fiber-like structure, exhibited the amide III band characteristic of the β-sheet structure, as confirmed by FTIR. This configuration contributed to the structural reinforcement, leading to the slow degradation rate of the fabricated membrane [45]. During the degradation of PVA components, the pore size of the membranes might have increased and facilitated angiogenesis and nutrient supply into the grafted site, while the BCP degraded, leading to calcium release, and induced bone formation.

#### 5.1.8. The Calcium Release Profile of the Membranes

**Figure 9:**
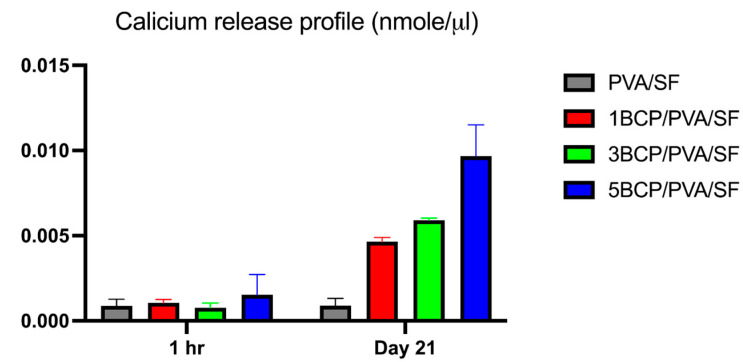
The cumulative calcium release profile of the fabricated membranes in ultra-pure water at 1 h and on day 21.

The cumulative calcium release profile from the fabricated membranes in ultra-pure water is demonstrated in Figure 9. On day 21, calcium contents were released from 1BCP/PVA/SF, 3BCP/PVA/SF, and 5BCP/PVA/SF membranes at 0.005 ± 0.0002, 0.0059 ± 0.0001, and 0.01 ± 0.002 nmole/µL, respectively. Moreover, the calcium ions’ release capability may actively promote osteoblast proliferation and bone regeneration.

### 5.2. The Mechanical Characterization of the Semi-Resorbable Bioactive Barrier Membranes

**Figure 10:**
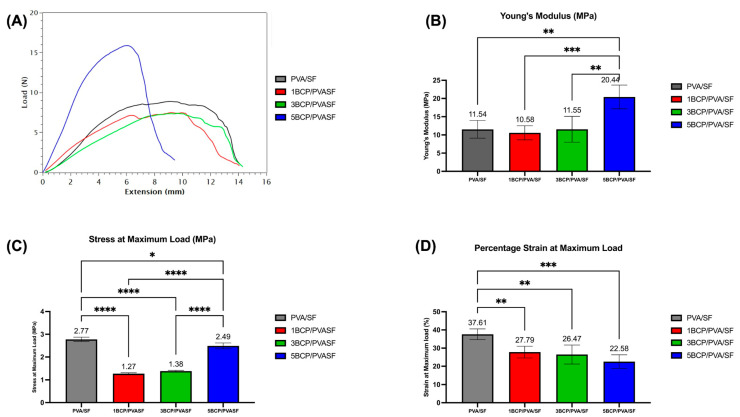
Tensile strength testing of the fabricated membranes: (**A**) load–extension graph, (**B**) Young’s modulus, (**C**) mean stress at maximum load, and (**D**) mean strain at maximum load. (* = significant difference between groups (*p* ≤ 0.05), ** = significant difference between groups (*p* ≤ 0.01), *** = significant difference between groups (*p* ≤ 0.0001), and **** = significant difference between groups (*p* ≤ 0.00001)).

The mechanical properties of the barrier membranes were inspected and are shown in Figure 10.

The 5BCP/PVA/SF membrane showed lower elasticity than other groups and also possessed the highest Young’s modulus (20.44 ± 3.26 MPa), which was significantly different (*p* < 0.01) from PVA/SF (11.54 ± 2.42 MPa), 1BCP/PVA/SF (10.58 ± 1.94 MPa), and 5BCP/PVA/SF (11.55 ± 3.54 MPa).

The PVA/SF membrane showed the highest stress at maximum load (2.77 ± 0.09 MPa), followed by 5BCP/PVA/SF (2.49 ± 0.13 MPa), which was higher than the dried Porcine Bone Lamina (2.1 MPa), while the 3BCP/PVA/SF (1.37 ± 0.33 MPa) and 1BCP/PVA/SF (1.27 ± 0.37 MPa) values were less than the dry Porcine Tendon (1.8 MPa) and dry Equine Bone Lamina (1.7 MPa), respectively [46].

The percentage of strain was highest in PVA/SF (37.61 ± 02.95%), which was significantly different (*p* < 0.01) from 1BCP/PVA/SF (27.79 ± 3.22%), 3BCP/PVA/SF (26.47 ± 5.23%), and 5BCP/PVA/SF (22.58 ± 3.73 MPa).

For effective guided bone regeneration, the membrane should combine adequate mechanical strength, to resist compression from surrounding tissues, with sufficient flexibility, for convenient handling, contouring, and adaptation to the intended defect morphology [2,47]. From these points, 1BCP/PVA/SF and 3BCP/PVA/SF are not too rigid, are comparable to the tendon, and can still be stretched.

### 5.3. The Biological Characterization of the Semi-Resorbable Bioactive Barrier Membrane Cell Attachment and Morphology

**Figure 11:**
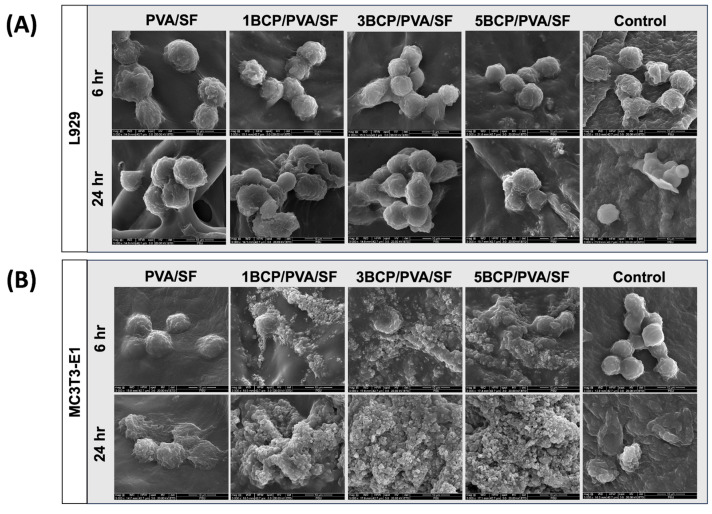
SEM images of cell attachment at magnification of ×3000: (**A**) fibroblast cell attachment on the top surface of the membranes at 6 and 24 h and (**B**) osteoblast cell attachment on the bottom surface of the membranes at 6 and 24 h.

Cells were cultured on the membranes for 6 and 24 h. The SEM images showed that the L929 cells and the MC3T3-E1 cells were well attached onto the material surface (Figure 11).

At 6 h, the fibroblast cells could attach to the membrane, and at 24 h, the cell numbers increased and started to spread on the membranes in a similar pattern in all groups. For the osteoblast cells, cell attachment could be found on the membranes at 6 h. After 24 h, the cell numbers increased and started to spread on the surface of the membranes, particularly in the BCP-incorporated membrane, which showed numerous cells aggregated on the membranes. It can be implied that the BCP incorporation enhanced the osteoblast cell attachment.

In summary, the semi-resorbable barrier membranes derived from silk fiber sheets were successfully fabricated, resulting in a flexible white membrane with side-specific properties according to the precipitation of BCP during the fabrication process on the bottom surface and the designation to face the bone. All membranes were porous, possessed barrier functions with good mechanical properties, were rigid enough to maintain space, and could be stretched to adapt to the grafted site. The semi-resorbable property of the membranes enhanced the vascularization and nutrient supply during the degradation of PVA components. For the BCP-incorporated membrane, which showed numerous aggregated osteoblast cells, it can be inferred that the BCP degradation led to calcium release and enhanced osteoblast attachment and later bone formation. Further studies on the in vitro and in vivo biological properties, bioactivity, and barrier function of the fabricated membranes should be conducted to confirm the properties of the membranes.

## 6. Conclusions

In this study, novel semi-resorbable bioactive barrier membranes derived from silk fiber sheets were successfully fabricated, and 3BCP/PVA/SF was identified as the most promising formulation. The addition of PVA improved the physical properties of the fabricated membrane by enhancing hydrophilicity, while the BCP had the potential to promote bone formation. Following the degradation of the PVA binder, allowing angiogenesis and nutrient exchange, the incorporated BCP was expected to induce bone formation and ultimately integrate with the surrounding tissue. The semi-resorbable bioactive barrier membrane based on silk fiber sheets demonstrated physicochemical, mechanical, and structural properties suitable for maintaining space during the bone regeneration process in GBR procedures.

## Figures and Tables

**Table 1:** Experimental groups.

Barrier Membrane	Percentages of BCP (*w*/*v*) in PVA Suspension	Abbreviation
PVA/silk fiber sheet	0	PVA/SF
1% BCP in PVA suspension/silk fiber sheet	1% BCP in PVA suspension	1BCP/PVA/SF
3% BCP in PVA suspension/silk fiber sheet	3% BCP in PVA suspension	3BCP/PVA/SF
5% BCP in PVA suspension/silk fiber sheet	5% BCP in PVA suspension	5BCP/PVA/SF
Commercial collagen membrane (Ossix^®^ Plus)	-	Control

## Data Availability

The original contributions presented in this study are included in the article. Further inquiries can be directed to the corresponding author.
